# Formation of reactive oxygen species by irradiation of cold atmospheric pressure plasma jet to water depends on the irradiation distance

**DOI:** 10.3164/jcbn.18-102

**Published:** 2019-03-07

**Authors:** Kazunori Anzai, Tamami Aoki, Satoko Koshimizu, Reina Takaya, Kazunori Tsuchida, Tokuko Takajo

**Affiliations:** 1 Faculty of Pharmaceutical Sciences, Nihon Pharmaceutical University, 10281 Komuro, Ina-machi, Kitaadachi-gun, Saitama 362-0806, Japan

**Keywords:** cold atmospheric pressure plasma jet, hydroxyl radical, Fricke dosimeter, hydrogen peroxide, ESR

## Abstract

Because application of cold atmospheric pressure plasma jet (CAPPJ) to biological samples have taken large attentions, it is important to examine the effects of various CAPPJ parameters on the generation of reactive species. Here, we investigated the generation of reactive species in water by CAPPJ irradiation by changing the following parameters: irradiation time, sample volume, and irradiation distance between the sample surface and plasma jet tip. We measured 1) change in the ESR signal intensity of 4-hydroxy-2,2,6,6-tetrametylpeperidine-1-oxyl (Tempol), 2) spin-trapping with 5,5-dimethyl-1-pyrroline *N*-oxide (DMPO), 3) Fricke dosimeter reaction, and 4) hydrogen peroxide (H_2_O_2_) formation induced by CAPPJ irradiation. By the experiment of volume dependency, it is suggested that the reactive species detected in water are formed largely in the plasma gas phase. The reduction of ESR signal intensity of Tempol and the formation of DMPO-OH were strongly dependent on irradiation distance, but the relationship between H_2_O_2_ generation and distance was weak. The formation of species that oxidize Fe^2+^ to Fe^3+^ was shown by the Fricke dosimeter reaction, and reactions at irradiation distances longer than 3 cm were mainly attributable to H_2_O_2_. It may be possible to apply different reactive species to the samples by changing the CAPPJ irradiation distance.

## Introduction

The development of plasma technology enables the generation of cold atmospheric plasma (CAP).^(1)^ CAP can be applied to biological samples because it is produced at room temperature and normal atmospheric pressure.^(2)^ Therefore, CAP has potential for biomedical applications such as wound healing,^(3–6)^ infection control,^(7–10)^ and cancer therapy.^(11–14)^ There are at least three types of CAP used for biological samples: 1) plasma jets, 2) corona discharge plasma sources, and 3) dielectric barrier discharge plasma sources.^(4)^ Plasma jets are easy to handle and convenient to apply to *in vivo* samples, such as mice and humans, in addition to *in vitro* samples such as cell suspensions and membrane vesicles. Various reactive species are produced by irradiation of cold atmospheric pressure plasma jet (CAPPJ) to water.^(15–18)^ However, because there are many parameters that affect CAPPJ properties, such as the content of carrier gas, the flow rate of carrier gas, irradiation time, and irradiation distance, it is important to reveal the effects of each parameter in detail for practical applications of CAPPJ to biological samples. Here, we used CAPPJ with helium as a carrier gas and examined the effect of the distance between the plasma jet nozzle and the water surface of the sample on the generation of reactive species in the solution.

## Materials and Methods

### Chemicals

Ultrapure helium gas (>99.999%) was obtained from Saisan Co., Ltd. (Saitama, Japan). 5,5-dimethyl-1-pyrroline *N*-oxide (DMPO) was obtained from Labotec Co., Ltd., Tokyo, Japan. 4-hydroxy-2,2,6,6-tetrametylpeperidine-1-oxyl (Tempol) was obtained from Sigma Co., Ltd., (St. Louis, MO). Other reagents were obtained from Wako Pure Chemical Industries, Ltd. (Osaka, Japan). All the reagents were analytical grade and used without further purification. Ultrapure water (Milli-Q water) was prepared with a MiIli-Q Gradient A10 water purification system (Millipore Co., Ltd., Billerica, MA).

### Irradiation of cold atmospheric pressure plasma jet (CAPPJ)

**Fig. 1:**
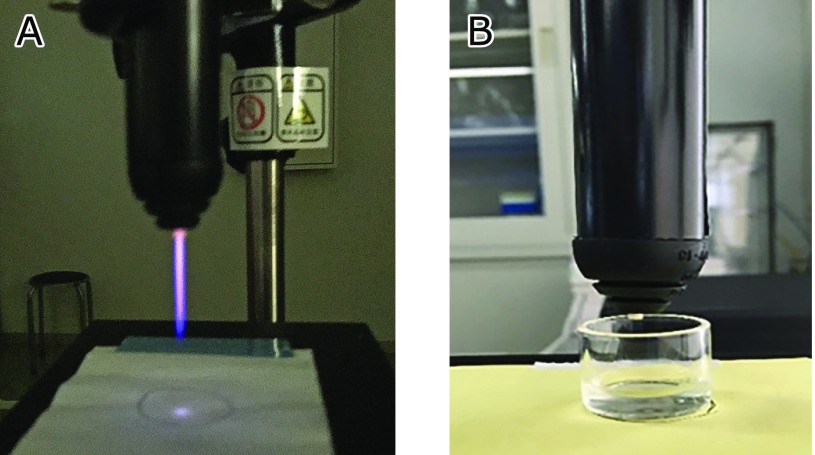
Plasma jet (A) and the setup for the irradiation of plasma to the water sample (B). The length of the plasma jet was about 3 cm in our experimental conditions. The sample vessel for irradiation was made of glass with a 20-mm inside diameter and 20-mm height.

CAPPJ was generated using a plasma head (TPN-20, NU Global, Nagoya, Japan) and an electric regulating unit (PN-110TPG, NU Global) with helium as a carrier gas. The flow rate of the helium gas was regulated by a mass flow controller (CUBE GM2, Fcon Co., Ltd., Nankoku, Kochi, Japan), and the value was set at 5 L/min. The length of the plasma jet was about 30 mm (Fig. 1A). CAPPJ was applied to an aqueous sample in a vessel made of glass with a 20-mm inside diameter and 20-mm height (Fig. 1B). The sample volume was set at 1 ml unless otherwise stated, and the parameters of the irradiation time, sample volume, and the irradiation distance between the surface of the sample solution and the plasma jet tip varied.

### ESR measurement

The change in the ESR signal intensity of 0.01 mM Tempol by CAPPJ irradiation was measured with an ESR spectrophotometer (JES-FA100, JEOL, Akishima, Tokyo, Japan) using a quartz flat cell. MnO_2_ was used as the external standard for ESR measurement. The relative intensity of the first of three lines of Tempol with respect to the intensity of the MnO_2_ signal was used for quantitative analysis.

For ESR spin-trapping experiments, DMPO was used as a spin-trap reagent. An aqueous solution of DMPO (10 mM, 1 ml) was irradiated by CAPPJ, and the resultant solution was transferred to the quartz flat cell immediately. At 2 min after the end of the irradiation, ESR spectra were measured in the presence of MnO_2_ as an external standard.

### Fricke dosimeter

Formation of oxidative species by CAPPJ irradiation was evaluated using the Fricke dosimeter solution. The Fricke dosimeter solution was prepared by dissolving 3.920 g of ammonium iron (II) sulfate (Mohr’s salt), 0.015 g of sodium chloride, and 5.6 ml of sulfonic acid in Milli-Q water to 250 ml.^(19)^ After the Fricke dosimeter solution (1 ml) was irradiated with CAPPJ, the solution was diluted to 3 ml by adding Milli-Q water, and the absorbance at 304 nm was measured with a UV-visible spectrophotometer (UV-2550, Shimadzu, Kyoto, Japan).

### Measurement of hydrogen peroxide

Formation of hydrogen peroxide (H_2_O_2_) by CAPPJ irradiation was measured by the colorimetric method using a peroxidase reaction according to a previously reported method with slight modifications.^(20)^ The colorimetric reagent solution (100 ml) contained 0.234 g of phenol, 0.10 g of 4-aminoantipyrine, 1 ml of 0.1 M phosphate buffer (pH 7.0), and 0.24 mg horse radish peroxide (Wako Pure Chemical Industries, Ltd.). After irradiation, 2 ml of reagent solution was added to 1 ml of the sample solution, and the absorbance at 505 nm of the mixture was measured with a UV-visible spectrophotometer.

## Results

### Change in the ESR signal intensity of Tempol by CAPPJ irradiation

**Fig. 2:**
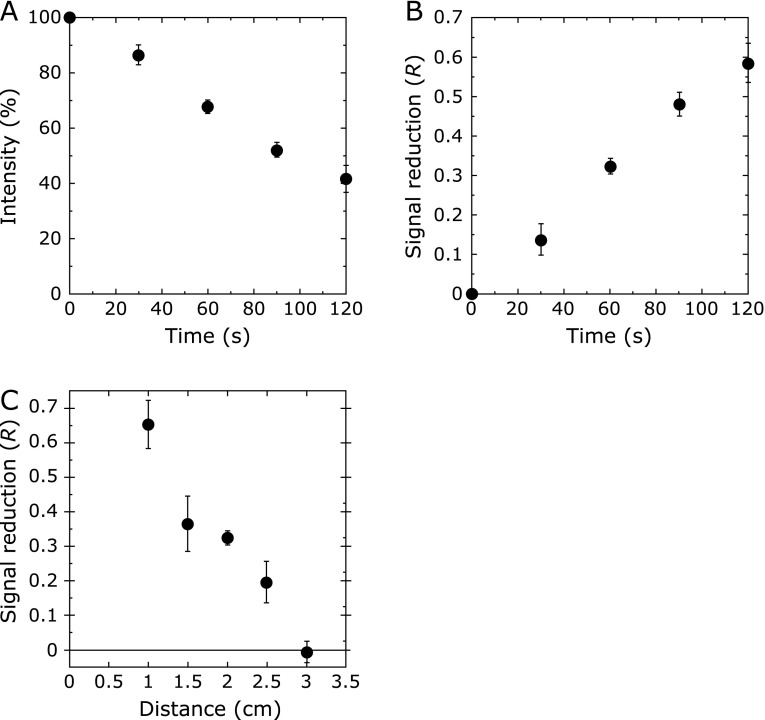
Change in the signal intensity of Tempol by CAPPJ irradiation. (A) The time-dependent change based on irradiation. Tempol solution (0.01 mM, 1 ml) was irradiated at a distance of 2 cm. The signal intensity was plotted as a percentage of the signal intensity compared to that of no irradiation. Error bar shows SD (*n* = 3). (B) The signal intensity was converted to the signal reduction ratio (*R*), and the *R* value was plotted vs irradiation time. The *R* was defined as *R* = (*I*_0_ – *I*) / *I*_0_, where *I*_0_ and *I* were the intensity obtained by no irradiation and irradiation, respectively. Error bar shows SD (*n* = 3). (C) The relationship between signal reduction ratio (*R*) and irradiation distance. Tempol solution (0.01 mM, 1 ml) was irradiated for 60 s by varying the irradiation distance from 1.0 to 3.0 cm. Error bar shows SD (*n* = 3).

We used the reaction of a stable nitroxide radical Tempol with other free radicals. A Tempol solution (0.01 mM) was irradiated with CAPPJ, and the change in the ESR signal intensity versus irradiation time was measured with a fixed irradiation distance (2 cm). The signal intensity of Tempol decreased depending on the increase in the plasma irradiation time (Fig. 2A), suggesting that Tempol was converted to a non-radical form by the reaction with the reactive species generated by CAPPJ irradiation. We defined the signal reduction ratio (*R*) as *R* = (*I*_0_ – *I*) / *I*_0_, where *I*_0_ and *I* were the intensity obtained by no irradiation and irradiation, respectively, and the result was re-plotted with *R*. As shown in Fig. 2B, the *R* value increased with irradiation time. When irradiation distance between the solution surface and the plasma jet nozzle was changed with fixed irradiation time (60 s), the *R* value decreased with the increase in irradiation distance and reached almost 0 at a distance of more than 2.5 cm (Fig. 2C). The distance of 2.5 cm corresponded to the length of the plasma jet.

### Observation of spin trapped radicals

**Fig. 3:**
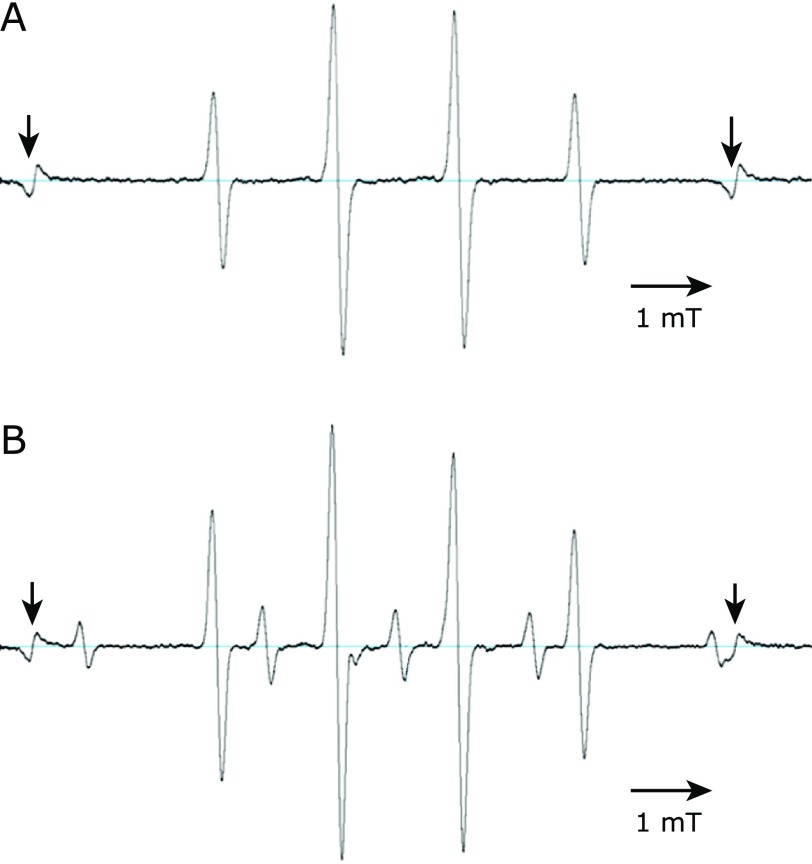
ESR spectra obtained after plasma irradiation of DMPO solution. DMPO solution (10 mM) was irradiated with the plasma for 30 s at a distance of 1.5 cm (A) or 0.5 cm (B). The irradiated solution was transferred to a quartz flat cell immediately, and ESR spectra were measured 2 min after the end of the irradiation. MnO_2_ signal (arrows) is shown for the external standard.

Several reactive species were generated by CAPPJ irradiation of water. Next, we focused on the generation of ^•^OH using a spin trapping technique with DMPO as a spin trap. When DMPO solution (1 ml) was irradiated for 30 s with CAPPJ at an irradiation distance of 1.5 cm, a typical DMPO-OH signal was observed (Fig. 3A), indicating the existence of ^•^OH in the solution. When the DMPO solution was irradiated at an irradiation distance of 0.5 cm, a different spectrum was observed (Fig. 3B). The signal pattern was similar to the ESR signal observed by X-irradiation or carbon ion irradiation to DMPO solution.^(21)^ DMPO-H signal was observed overlapping the DMPO-OH signal (Fig. 3B). This DMPO-H signal was observed only when irradiation was occurred a distance of 1 cm or less.

**Fig. 4:**
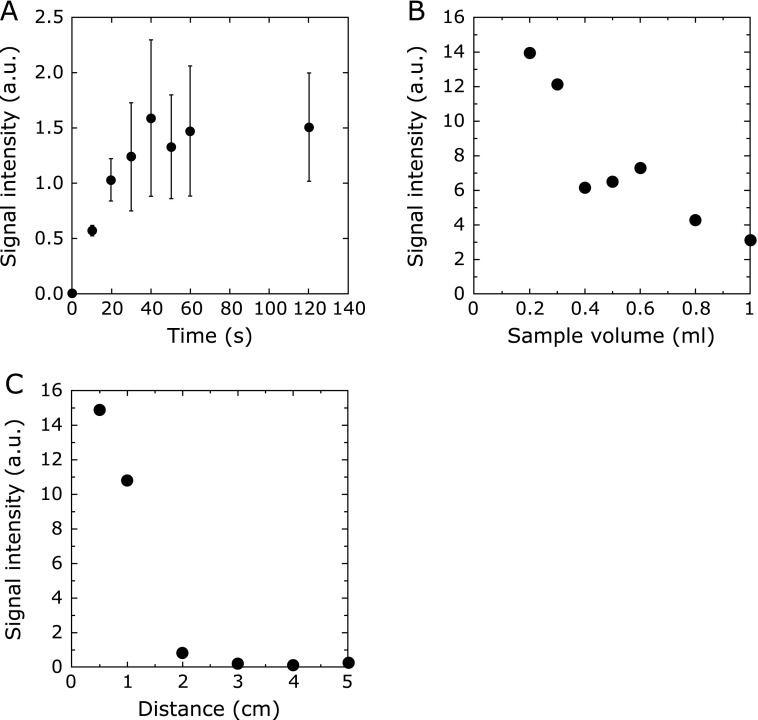
Change in the DMPO-OH signal intensity by CAPPJ irradiation. (A) Irradiation time dependency. DMPO solution (10 mM, 1 ml) was irradiated with CAPPJ at a distance of 1.5 cm by changing the irradiation time. After the irradiation, the solution was transferred to a quartz flat cell immediately, and ESR spectra were measured 2 min after the end of the irradiation. Error bar shows SD (*n* = 3). (B) The relationship between DMPO-OH signal intensity and sample volume. DMPO solution (10 mM) with a different volume was irradiated with CAPPJ for 30 s at a distance of 1.5 cm. After the irradiation, the solution was transferred to a quartz flat cell immediately, and ESR spectra were measured 2 min after the end of the irradiation. (C) The relationship between the signal intensity of DMPO-OH and irradiation distance. DMPO solution (10 mM, 1 ml) was irradiated with the plasma for 30 s by varying the irradiation distance from 0.5 to 5 cm. After the irradiation, the solution was transferred to a quartz flat cell immediately, and ESR spectra were measured 2 min after the end of the irradiation.

When the DMPO solution (1 ml) was irradiated at an irradiation distance of 1.5 cm by changing the irradiation time, the intensity increased with time and reached plateau at about 40 s (Fig. 4A).

When a different volume of DMPO solution (10 mM, 0.2–1 ml) was irradiated by CAPPJ for 30 s at an irradiation distance of 1.5 cm, the signal intensity of DMPO-OH decreased with the increase in the volume of the DMPO solution (Fig. 4B). By increasing the volume from 0.2 ml to 1.0 ml, the signal intensity decreased inversely to about 1/5 of the signal observed for a sample of 0.2 ml.

When the DMPO solution (1 ml) was irradiated for 30 s with CAPPJ by varying the irradiation distance from 0.5 to 5 cm, the signal intensity decreased as the distance increased (Fig. 4C). No signal was observed at a distance of more than 3 cm.

### Oxidation reaction of the Fricke dosimeter solution by plasma irradiation

**Fig. 5:**
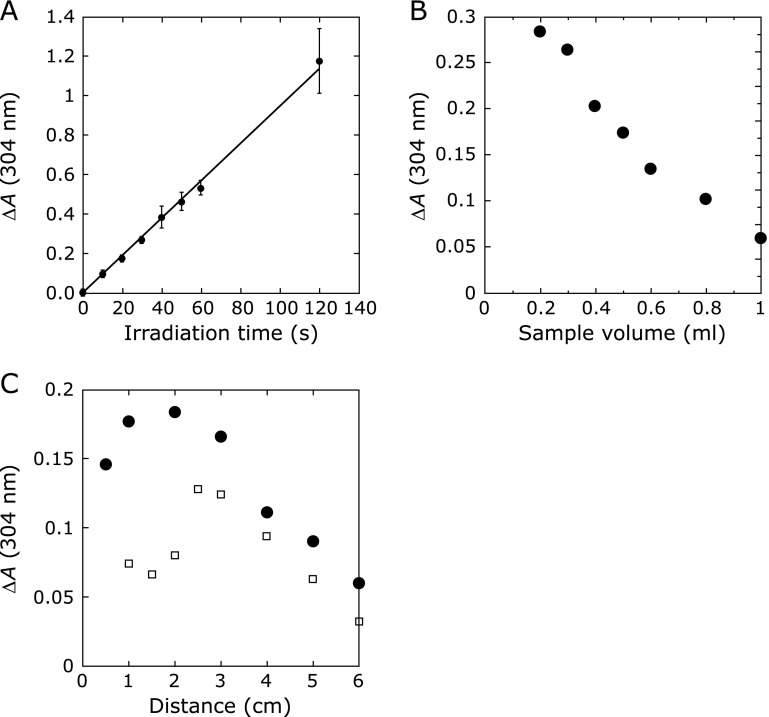
Change in the absorbance of Fricke dosimeter solution by CAPPJ irradiation. (A) Irradiation-time dependent increase in the absorbance of the Fricke dosimeter solution. The Fricke dosimeter solution (1 ml) was irradiated with CAPPJ at a distance of 1.5 cm. The irradiated solution was diluted with 2 ml of water and the absorbance at 305 nm was measured. Error bar shows SD (*n* = 3). (B) Effect of sample volume on the reaction of the Fricke dosimeter solution with the plasma. The Fricke dosimeter solution of 0.2–1.0 ml was irradiated with CAPPJ for 30 s at a distance of 1.5 cm. After the irradiation, 0.15 ml of each sample was diluted to 2.4 ml by adding water, and the absorbance at 304 nm was measured. (C) Effect of irradiation distance on the reaction of the Fricke dosimeter solution. Closed circle (●) shows the absorbance change at 304 nm of the Fricke dosimeter solution by CAPPJ irradiation. The Fricke dosimeter solution (1 ml) was irradiated with CAPPJ for 30 s by varying the irradiation distance from 0.5 to 6 cm. The irradiated solution was diluted with 2 ml of water, and the absorbance at 304 nm was measured. Open square (□) shows the estimation of the contribution of H_2_O_2_ on the absorbance change using data from Fig. 6B and Fig. 7.

The Fricke dosimeter measures the oxidation of Fe^2+^ to Fe^3+^ by oxidizing species, such as a hydroxyl radical (^•^OH), produced by ionizing radiation. Because irradiation of atmospheric plasma generates various oxidizing species in water,^(15)^ we investigated the oxidation reaction by CAPPJ irradiation. The change in the absorbance at 304 nm of the Fricke dosimeter solution by CAPPJ irradiation was measured as follows. The Fricke dosimeter solution (1 ml) was irradiated with CAPPJ at an irradiation distance of 1.5 cm. The resultant irradiated solution was diluted with 2 ml of water, and the absorbance at 304 nm was measured. The absorbance at 304 nm increased linearly in proportion to the irradiation time (Fig. 5A).

To examine whether reactive species were produced homogeneously in solution by a reaction with CAPPJ irradiation or were produced inhomogeneously only at the surface of the solution, the sample volume was changed. A different volume of the Fricke dosimeter solution (0.2–1.0 ml) was used to evaluate the oxidation reaction by CAPPJ irradiation. After irradiation for 30 s, 0.15 ml of each sample was diluted to 2.4 ml by adding 2.25 ml of water, and the absorbance at 304 nm was measured. The absorbance change decreased with an increase in the sample volume (Fig. 5B). The absorbance change of 1.0 ml sample was about 1/5 of that of the 0.2 ml sample.

The distance between the solution surface and the plasma jet nozzle must be an important parameter because the reaction took place only at the solution surface as shown above. Therefore, we next examined the effect of irradiation distance on the oxidation reaction of the Fricke dosimeter solution. Figure 5C shows that the typical relationship between the oxidation reaction and the irradiation distance varied from 0.5 to 6 cm when the irradiation time was fixed at 30 s. The figure shows that the maximum oxidation reaction was at an irradiation distance of around 1–2 cm. The oxidation reaction decreased significantly at a distance over 2 cm, but more than 25% of the maximum change was still observed even at a distance of 6 cm.

### H_2_O_2_ formation by plasma irradiation

**Fig. 6:**
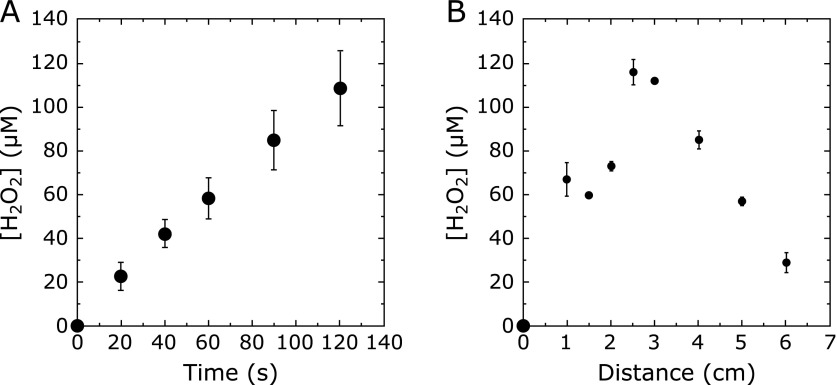
Change in the concentration of hydrogen peroxide generated by CAPPJ irradiation. (A) Irradiation time dependency. Pure water (1 ml) was irradiated with CAPPJ at a distance of 1.5 cm. After the irradiation, 2 ml of 4-aminoantipyrine/phenol solution was added to each sample, and the absorbance at 505 nm was measured. Error bar shows SD (*n* = 4). (B) The relationship between H_2_O_2_ formation and irradiation distance. Pure water (1 ml) was irradiated with CAPPJ for 60 s at various distances from 1 to 6 cm. Error bar shows SD (*n* = 3).

Water (1 ml) was irradiated with CAPPJ for 20–120 s at an irradiation distance of 1.5 cm, H_2_O_2_ formation was quantified, and the concentration of H_2_O_2_ increased almost linearly with time (Fig. 6A). When water (1 ml) was irradiated with CAPPJ for 60 s by changing the irradiation distance from 1.0 to 6.0 cm, the relationship between H_2_O_2_ formation and irradiation distance was not strong, and a considerable amount of H_2_O_2_ was still observed even at an irradiation distance of 6.0 cm (Fig. 6B).

### Effect of H_2_O_2_ on the oxidation reaction of the Fricke dosimeter solution

**Fig. 7:**
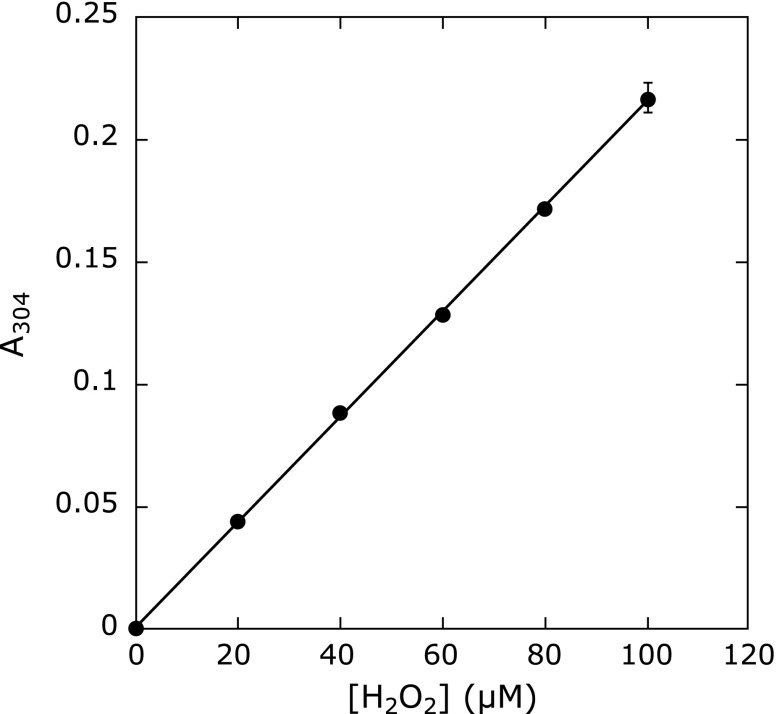
Effect of H_2_O_2_ on the absorbance change of Fricke dosimeter solution. H_2_O_2_ was added to the Fricke dosimeter solution, and the absorbance change at 304 nm was measured. Error bar shows SD (*n* = 3).

Since it was found that H_2_O_2_ is produced by the irradiation of CAPPJ, the formation of H_2_O_2_ may cause some contribution to the reaction of the Fricke dosimeter reaction. Therefore, we examined the effect of H_2_O_2_ on the oxidation reaction of the Fricke dosimeter solution. As shown in Fig. 7, H_2_O_2_ oxidizes Fe^2+^ to Fe^3+^ in the Fricke dosimeter solution.

## Discussion

We have investigated the generation of reactive species in water by CAPPJ irradiation by measuring 1) spin-trapping adducts, 2) change in the intensity of the Tempol signal, 3) the Fricke dosimeter reaction, and 4) H_2_O_2_ formation, and we examined the effects of irradiation time and irradiation distance.

The Tempol signal intensity decreased almost linearly with irradiation time. We previously investigated the reaction of stable nitroxide radicals with reactive oxygen species, such as ^•^OH, O_2_, and ^1^O_2_, and reported that ESR signal intensity of stable nitroxide radicals decreases by the reaction with ^•^OH.^(22)^ Therefore, the signal reduction of Tempol may be derived by the reaction of Tempol with reactive oxygen species, most likely ^•^OH. The reduction of Tempol signal intensity was dependent on irradiation distance; no reduction was observed at more than 3 cm.

Next, we examined the formation of ^•^OH by a spin trapping technique. Formation of DMPO-OH was observed, which confirms that ^•^OH was produced in the irradiated solution. When the irradiation time was changed with a constant irradiation distance (1.5 cm), the intensity of the DMPO-OH signal increased at first with time but the velocity decreased gradually and the intensity reached plateau after 40 s. By spin trapping ^•^OH, DMPO became DMPO-OH with a nitroxide radical structure. Since the DMPO-OH radicals lose their intensity by a radical-radical reaction, this is one possible explanation for the observation of the plateau. Additionally, it is plausible that the nitroxide structure of DMPO-OH was converted to the non-radical form by reaction with reactive species produced by CAPPJ irradiation as was observed in the above experiments where the signal intensity of Tempol decreased by CAPPJ irradiation.

The spin trapping of ^•^OH was dependent on irradiation distance; no signal was observed at more than 3 cm. The relationship between DMPO-OH formation and irradiation distance was also reported by Uchiyama *et al.*^(16)^ DMPO-H formation was also dependent on irradiation distance, and the DMPO-H signal was observed only at a distance equal to 1 cm or less. This finding suggests that the ^•^OH and ^•^H radicals formed in the plasma jet reaches only 3 cm and 1 cm, respectively, from the plasma tip in our experimental conditions. This length of 3 cm corresponds to the length of the plasma jet. The difference of the distance-dependency between DMPO-OH and DMPO-H may be caused by the difference in the lifetime of ^•^OH and ^•^H.

In the Fricke dosimeter reaction, oxidation of Fe^2+^ to Fe^3+^ decreased with an increase in the sample volume, and the amount of product after irradiation of the 1.0 ml sample was about 1/5 of that of the 0.2 ml sample. This finding suggests that the Fricke dosimeter reaction takes place inhomogeneously only at the surface of the sample. In experiments of DMPO-OH formation, a similar relationship on sample volume was observed, which suggests that ^•^OH formation occurred only at the surface of the sample. Gorbanev *et al.*^(15)^ suggested that the reactive species detected in the liquid samples are formed largely in the plasma gas phase. The results of our experiments with the Fricke dosimeter solution and DMPO-OH formation support this idea.

The result that the reaction of Tempol reduction and DMPO-OH formation did not occur at a distance longer than 3 cm was different from that obtained from the experiment with Fricke dosimeter solution, where a significant reaction still occurred at a distance longer than 3 cm. This finding indicates that at the longer irradiation distance the reactive species for the oxidation of Fe^2+^ in the Fricke dosimeter solution was different from the species responsible for the reaction with Tempol and DMPO-OH formation, which was likely ^•^OH. This means that the helium plasma gas more than 3 cm away from the plasma jet nozzle contained some species capable of oxidizing Fe^2+^, but it contained no species capable of reacting with Tempol. In other words, some species with a longer lifetime and non-effectiveness for the reaction with Tempol may exist in the plasma gas, which was responsible for the oxidation of Fe^2+^.

H_2_O_2_ is not a free radical, and the reactivity is relatively weak compared to that of other reactive oxygen species. However, H_2_O_2_ has an important role in signal transduction in biological systems. Irradiation of cold atmospheric plasma to aqueous samples generates H_2_O_2_ in the liquid phase.^(15,18,23)^ H_2_O_2_ is a key molecule responsible for anti-cancer, anti-microbial, and wound-healing reactions of CAP, but the detailed mechanisms are unknown. H_2_O_2_ formation also shows a similar relationship with the Fricke dosimeter reaction on the irradiation distance between the water surface and plasma tip nozzle. The peak of the H_2_O_2 _concentration was found at an irradiation distance around 2.5–3 cm, and the concentration gradually decreased over distances longer than 3 cm. However, the concentration of H_2_O_2_ was still 1/5 of the peak even at a distance of 6 cm. Because H_2_O_2_ can be formed from the combination of two ^•^OH molecules and the H_2_O_2_ is stable, the H_2_O_2_ observed in the irradiated water may be derived from H_2_O_2_ formed with ^•^OH molecules generated not in the water but in the plasma jet.

H_2_O_2_ at an acidic pH oxidizes Fe^2+^ to Fe^3+^. The similar relationship of H_2_O_2 _formation and the reaction of Fricke dosimeter solution on the irradiation distance suggests that the reaction of Fricke dosimeter solution by CAPPJ irradiation is mainly caused by the formation of H_2_O_2_ by CAPPJ irradiation. We measured the absorbance change in the Fricke dosimeter solution by addition of H_2_O_2_ (Fig. 7). From this result and the formation of H_2_O_2_ by CAPPJ irradiation, we calculated the contribution of H_2_O_2_ on the absorbance change at 304 nm in the Fricke dosimeter reaction (Fig. 5C). This estimation suggests that the Fricke dosimeter reaction observed by irradiation of CAPPJ at an irradiation distance longer than 3 cm occurs mainly because of the formed H_2_O_2_ by irradiation.

The findings in this paper suggest that although various reactive species are produced by CAPPJ, it may be possible to apply different reactive species selectively to samples by changing the irradiation distance of CAPPJ.

## Author Contributions

KA designed and supervised the study, obtained the funding, and drafted the manuscript. TT, TA, SK and RT collected the data. KT and TT contributed statistical analysis and critical revision of the manuscript.
